# Development and validation of a machine learning model to predict stroke risk based on the NHANES database

**DOI:** 10.1097/MD.0000000000045800

**Published:** 2025-11-07

**Authors:** Junzhang Huang, Wencai Liu

**Affiliations:** a Department of General Surgery, Lianjiang Traditional Chinese Medicine Hospital, Zhanjiang, Guangdong, China; b Department of Orthopedics, Shanghai Sixth People’s Hospital Affiliated to Shanghai Jiao Tong University School of Medicine, Shanghai, China.

**Keywords:** boruta algorithm, LASSO regression, machine learning, random forest, stroke

## Abstract

Stroke is a serious condition associated with high rates of incidence and mortality. Timely and accurate risk assessment is critical for improving prevention and clinical intervention. This study investigates the application of machine learning models in evaluating the risk of stroke. The data for this study were drawn from the National Health and Nutrition Examination Survey, covering the years 1999 to 2002. To construct predictive models and select relevant variables, 3 approaches were applied: LASSO regression combined with stepwise selection, random forest, and the boruta algorithm in conjunction with LASSO regression. Model performance was evaluated through multiple metrics, including receiver operating characteristic and precision–recall curves, as well as calibration and decision curve analyses. The study analyzed data from 9922 participants, among whom 358 had a history of stroke. Key predictors were identified through a combination of LASSO and stepwise regression, producing a model with strong discriminative performance (area under the curve [AUC] = 0.843). In comparison, the random forest approach selected fewer predictors and showed lower predictive accuracy (AUC = 0.612). A model developed using the boruta algorithm followed by LASSO regression achieved a similarly high level of performance (AUC = 0.828). These findings illustrate how different variable selection methods can influence the predictive accuracy of the resulting models. The machine learning model, which is created using the National Health and Nutrition Examination Survey database, and serves as a reliable means of predicting stroke risk. There is every possibility to set up accurate predictive models with the help of different variable selection techniques and modeling methods that have high accuracy and clinical value.

## 1. Introduction

Stroke, a sudden cerebrovascular disease, is a major threat to global public health with its high incidence and morbidity rates.^[1,2]^ If effective primary prevention measures are not taken soon, the burden of stroke worldwide may continue to rise, especially in economically underdeveloped countries.^[3]^ Hypertension and diabetes are the risk factors of stroke,^[4]^ and thus, early detection and prevention are very important in reducing the medical burden.

In recent years, the rapid development of machine learning technology has made its application in the healthcare field increasingly widespread, and machine learning has provided promising solutions for this field.^[5]^ While initial studies primarily concentrated on neuroimaging for data,^[6,7]^ the present approach emphasizes combining multiple data types to enhance diagnostic precision and dependability.

The National Health and Nutrition Examination Survey (NHANES) database is a nationwide survey study that collects a large amount of health and nutrition information about adults and children in the United States,^[8]^ providing a valuable data resource for the development of disease risk prediction models. While previous studies on stroke prediction have often relied on limited sample sizes, single-center data, or a narrow set of clinical indicators, the present study utilizes the nationally representative NHANES dataset, thereby improving the generalizability and robustness of the findings. Moreover, instead of employing only one modeling strategy, we applied and compared multiple advanced approaches for variable selection and model construction, including LASSO regression, stepwise regression, random forest, and the boruta algorithm. This comprehensive and integrative strategy not only highlights the most important predictors of stroke but also rigorously evaluates model performance across different methodologies. By addressing both the breadth of data and the depth of methodological comparison, our study offers clearer insights and more reliable tools for stroke risk prediction, providing practical advantages over prior research.

## 2. Methods

### 2.1. Research design

The aim of this study is to create and validate a machine-learning model that may be used in preventive healthcare for predicting the risk of having a stroke. The research follows a retrospective cohort design which makes use of the NHANES database as a means of obtaining the required data.

### 2.2. Data sources

This study comes from NHANES, a national health and nutrition survey project that provides comprehensive demographic, health, and lifestyle information. The data used in this study was sourced from NHANES conducted from 1999 to 2002. Excluding participants without stroke data and missing clinically significant variables, a total of 9922 participants were included in the analysis. The studies involving humans were approved by National Center for Health Statistics Research Ethics Review Board. The studies were conducted in accordance with the local legislation and institutional requirements. Participants gave informed consent to participate in the study before taking part.

### 2.3. Variable selection

The variables included in the study are: age, height, weight, body mass index, gender, race, hypertension, diabetes, asthma, arthritis, heart failure, coronary heart disease, angina pectoris, heart attack, emphysema, chronic bronchitis, liver conditions, cancer, and stroke.

### 2.4. Statistical analysis

All analyses were conducted using R software (version 4.4.1) and MSTATA online statistical platform, which is a medical statistical software developed by China Hongyu Technology Co., Ltd. Descriptive statistics were used to summarize baseline data. Continuous variables were expressed as mean ± standard deviation or median (interquartile range) as appropriate, and categorical variables as frequency and percentage. Group comparisons were performed using *t* tests, nonparametric tests, or Chi-square tests as appropriate. *P* < .05 was considered statistically significant. Advanced modeling and multivariate analyses, including data preprocessing, variable selection, model training, and performance evaluation, were also conducted in R.

### 2.5. Machine learning model building

This article adopts 3 methods of variable selection and model construction.^[1]^ LASSO regression + stepwise regression: variables were selected using LASSO regression, and the final model variables were determined through stepwise regression analysis.^[2]^ Random forest algorithm: the importance of variables was evaluated using the random forest algorithm, leading to the selection of key variables.^[3]^ Boruta algorithm + LASSO regression: important variables were first identified using the boruta algorithm, followed by model building with LASSO regression.

### 2.6. Model performance assessment

The article uses diverse methods to comprehensively appraise the performance of all models. The sequence is carried out visually by the use of a nomogram to display the contribution weight of each variable within the model. The receiver operating characteristic (ROC) curve and the area under the curve (AUC) are both used to compare the accuracy of the models used in discriminative ability. Precision–recall (PR) curve is useful in assessing how the models perform for diverse thresholds, particularly in the context of the imbalanced datasets. The calibration curves and clinical decision curve analysis are helpful in examining the accuracy of probability predictions and the potential clinical utility of the models.

## 3. Results

### 3.1. Baseline table of the patient data

This study included 9922 participants, of whom 358 had experienced a stroke. Clinical data such as age, height, weight, body mass index, gender, race, hypertension, diabetes, asthma, arthritis, heart failure, coronary heart disease, angina pectoris, heart attack, emphysema, chronic bronchitis, liver condition, and cancer were collected for statistical analysis. The baseline characteristics of participants’ data showed that there were statistically significant differences between the 2 groups in age, height, weight, gender, race, hypertension, diabetes, arthritis, heart failure, coronary heart disease, angina pectoris, heart attack, emphysema, chronic bronchitis, liver condition, and cancer (*P* < .05) (Table 1).

**Table 1:** Baseline table of participants.

Characteristic	Non-stroke, N = 9564*	Stroke, N = 358*	*P*-value
Age (years)	49 ± 19	70 ± 14	<.001†
Height (cm)	167 ± 10	164 ± 8	<.001†
Weight (kg)	77 ± 19	75 ± 19	.027†
BMI (kg/m^2^)	28.0 ± 5.9	28.4 ± 5.4	.169†
Gender			.016‡
Male	4430 (46.3%)	189 (52.8%)	
Female	5134 (53.7%)	169 (47.2%)	
Race			.002‡
Mexican American	4693 (49.1%)	198 (55.3%)	
Other Hispanic	1780 (18.6%)	80 (22.3%)	
Non-Hispanic White	2250 (23.5%)	62 (17.3%)	
Non-Hispanic Black	517 (5.4%)	10 (2.8%)	
Other race (including multi-racial)	324 (3.4%)	8 (2.2%)	
Hypertension			<.001‡
No	6865 (71.8%)	116 (32.4%)	
Yes	2699 (28.2%)	242 (67.6%)	
Diabetes			<.001‡
No	8714 (91.1%)	272 (76.0%)	
Yes	850 (8.9%)	86 (24.0%)	
Asthma			.988‡
No	8600 (89.9%)	322 (89.9%)	
Yes	964 (10.1%)	36 (10.1%)	
Arthritis			<.001‡
No	7335 (76.7%)	179 (50.0%)	
Yes	2229 (23.3%)	179 (50.0%)	
Heart failure			<.001‡
No	9319 (97.4%)	294 (82.1%)	
Yes	245 (2.6%)	64 (17.9%)	
Coronary heart disease			<.001‡
No	9226 (96.5%)	297 (83.0%)	
Yes	338 (3.5%)	61 (17.0%)	
Angina pectoris			<.001‡
No	9289 (97.1%)	301 (84.1%)	
Yes	275 (2.9%)	57 (15.9%)	
Heart attack			<.001‡
No	9216 (96.4%)	282 (78.8%)	
Yes	348 (3.6%)	76 (21.2%)	
Emphysema			<.001‡
No	9413 (98.4%)	331 (92.5%)	
Yes	151 (1.6%)	27 (7.5%)	
Chronic bronchitis			.005‡
No	9043 (94.6%)	326 (91.1%)	
Yes	521 (5.4%)	32 (8.9%)	
Liver condition			.001‡
No	9290 (97.1%)	337 (94.1%)	
Yes	274 (2.9%)	21 (5.9%)	
Cancer			<.001‡
No	8778 (91.8%)	289 (80.7%)	
Yes	786 (8.2%)	69 (19.3%)	

BMI = body mass index.

* Mean ± SD; n (%).

† Welch 2 Sample *t* test.

‡ Pearson Chi-squared test.

### 3.2. The LASSO regression + stepwise regression screened the variables and built a predictive model

**Figure 1.**
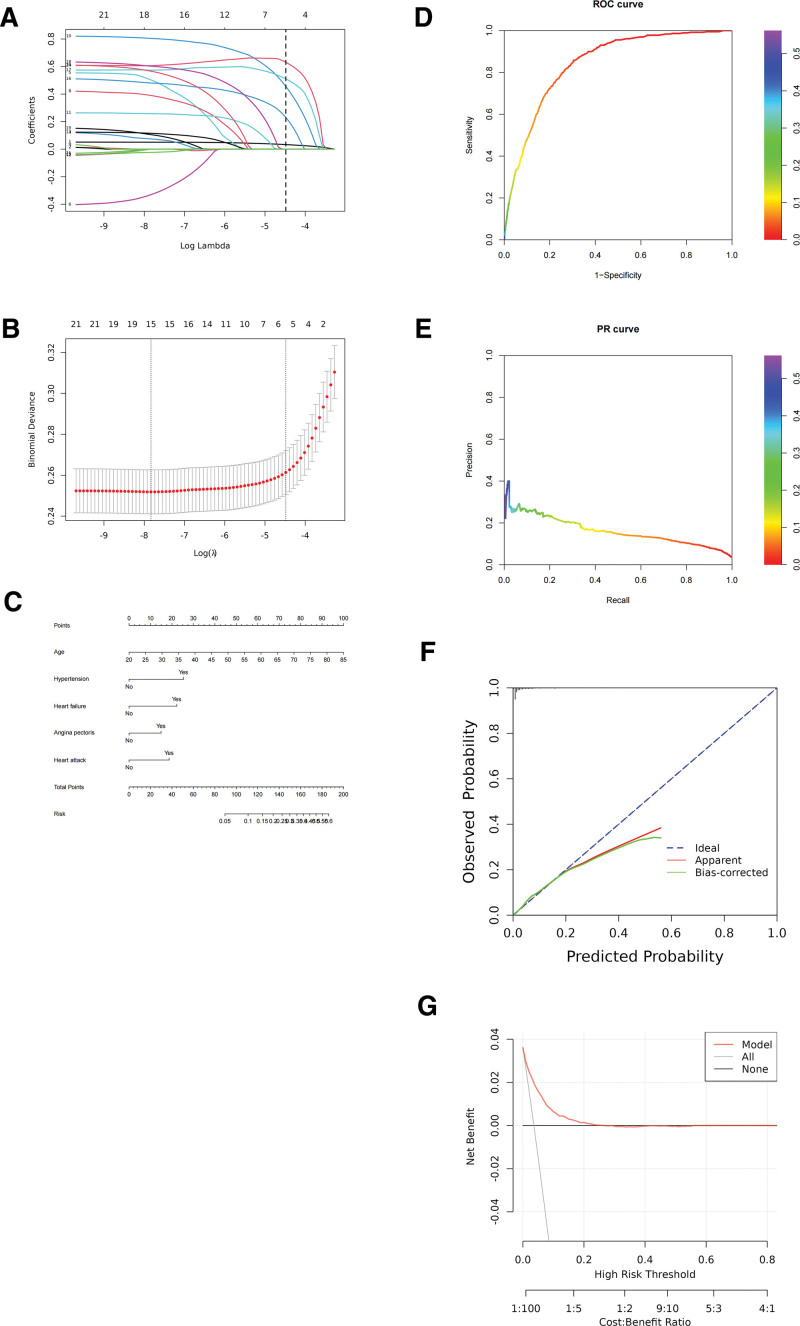
Process diagram for establishing the LASSO regression + stepwise regression model. (A) LASSO regression variable selection path diagram. (B) Cross-validation plot of LASSO regression. (C) Nomogram. (D) ROC curve. (E) PR curve. (F) Ideal calibration curve. (G) Clinical decision curve. PR = precision–recall, ROC = receiver operating characteristic.

A variable selection path diagram and a cross-validation plot were generated for the LASSO regression to illustrate the model’s variable selection process and optimal parameter tuning (Fig. 1A and B). Incorporating these selected variables into the original stepwise regression analysis, we found that age, hypertension, heart failure, heart attack, and angina pectoris showed statistically significant differences (*P* < .05) (Table 2). These variables were then used to develop a nomogram model (Fig. 1C). We plotted an ROC curve for analysis, along with a PR curve, yielding an AUC of 0.843 (Fig. 1D and E). Additionally, the calibration curve and clinical decision curve were utilized to assess the predictive model’s performance and patient benefit (Fig. 1F and G).

**Table 2:** Step-by-step regression table.

Characteristic	N	Event N	OR	95% CI	*P*-value
Age	9922	358	1.05	1.05–1.06	<.001
Hypertension					
No	6981	116	–	–	
Yes	2941	242	2.42	1.90–3.07	<.001
Heart failure					
No	9613	294	–	–	
Yes	309	64	2.16	1.53–3.06	<.001
Heart attack					
No	9498	282	–	–	
Yes	424	76	1.91	1.37–2.66	<.001
Angina pectoris					
No	9590	301	–	–	
Yes	332	57	1.68	1.17–2.40	.005

CI = confidence interval, OR = odds ratio.

### 3.3. Random forest was screened for variables and built predictive models

**Figure 2.**
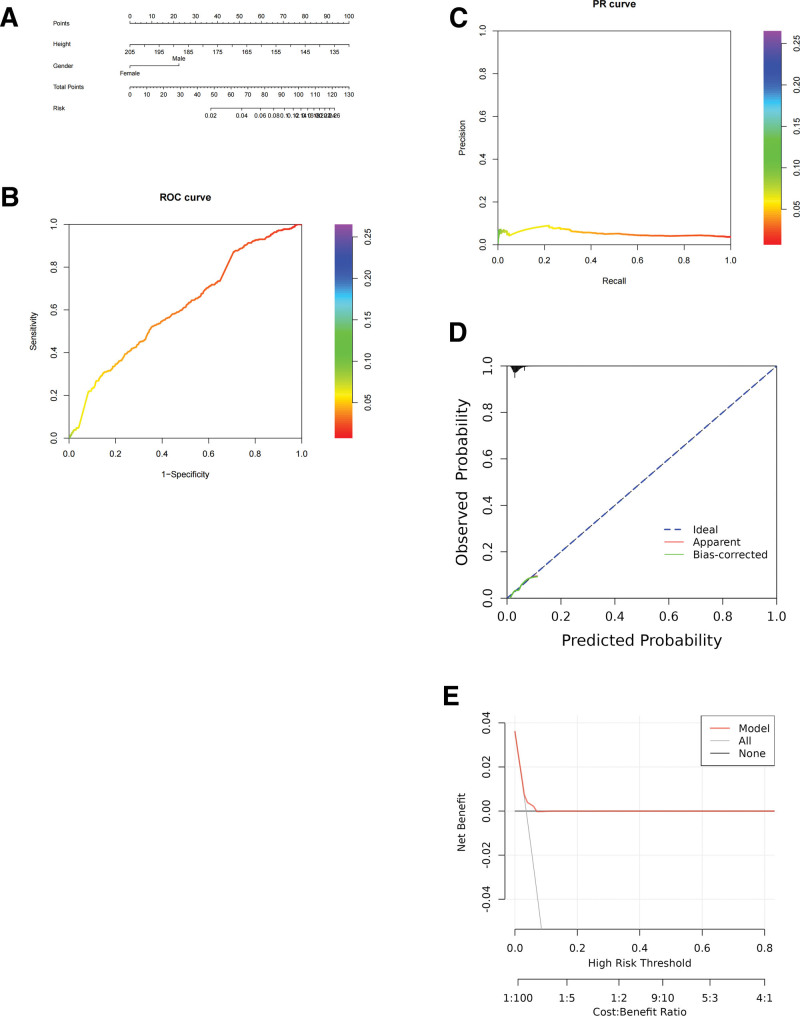
Flowchart for establishing random forest model. (A) Nomogram. (B) ROC curve. (C) PR curve. (D) Ideal calibration curve. (E) Clinical decision curve. PR = precision–recall, ROC = receiver operating characteristic.

A random forest model has been developed to both highlight the importance of each variable and quantitatively evaluate their individual contributions (Table 3). Establishing a column chart model using key variables selected by random forest model (Fig. 2A). ROC curve analysis and PR curve validation were conducted, resulting in an AUC of 0.612 (Fig. 2B and C). Additionally, calibration and clinical decision curves were plotted to evaluate the model’s performance (Fig. 2D and E).

**Table 3:** Random forest variable values.

Vars	Mean decrease accuracy	Mean decrease gini
Height	22.302273	107.661776
Gender	21.934391	17.551742
Weight	19.108240	116.042345
Heart failure	12.602382	15.997901
Coronary heart disease	11.066656	12.267619
Age	9.228669	105.446048
Angina pectoris	8.807770	13.104983
Arthritis	7.576430	16.413931
Race	6.819770	37.549033
Emphysema	6.548393	9.725320
Heart attack	4.587480	14.951205
Cancer	2.613936	14.252339
Chronic bronchitis	−1.515281	10.454079
Diabetes	−5.007375	14.505241
Liver condition	−8.412066	7.550695
Hypertension	−12.624467	16.624320

Mean decrease accuracy and mean decrease gini are 2 commonly used variable importance indicators in random forest to evaluate the contribution of each variable to the model’s predictive performance.

### 3.4. The variables were screened and the prediction model was built based on the boruta algorithm + LASSO regression

**Figure 3.**
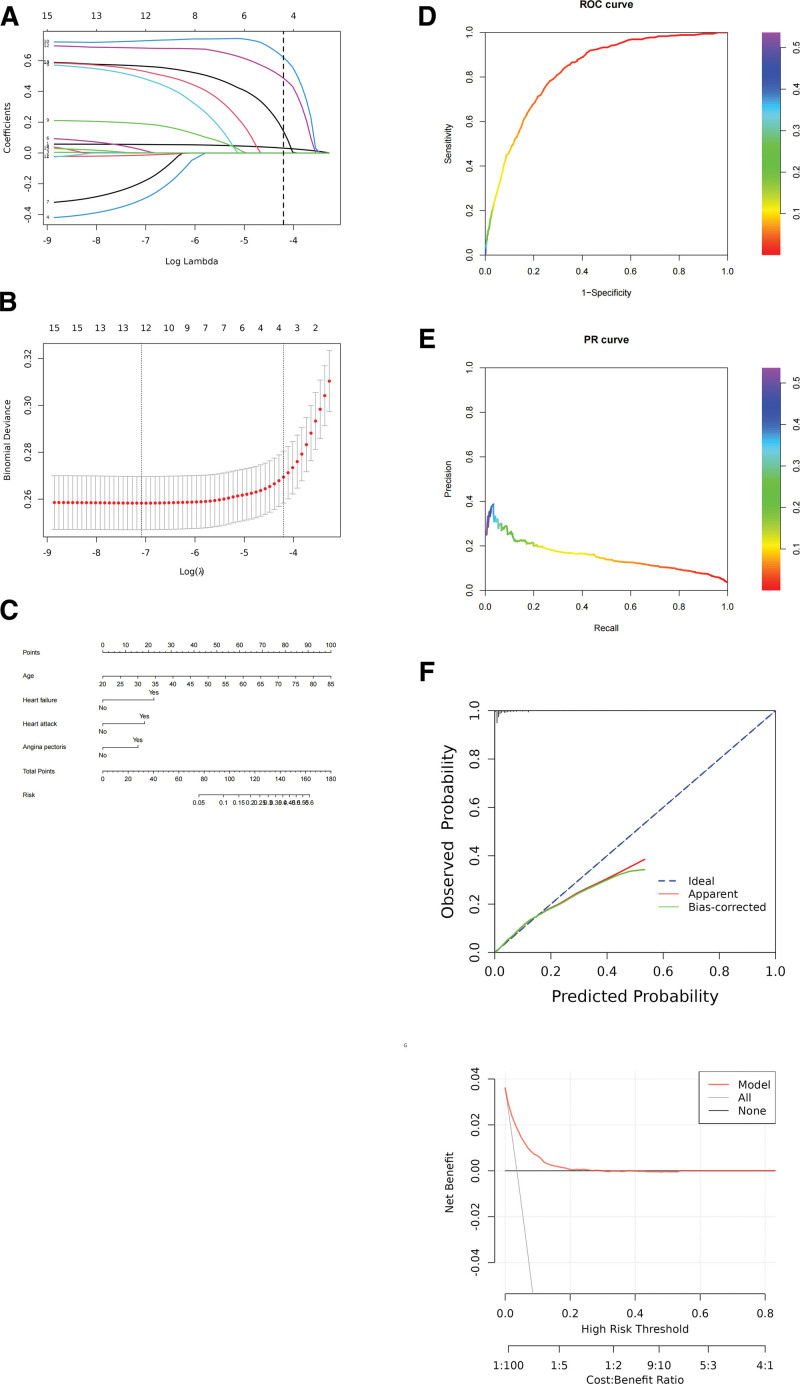
Flowchart for establishing boruta algorithm + LASSO regression model. (A) LASSO regression variable selection path diagram. (B) Cross-validation plot of LASSO regression. (C) Nomogram. (D) ROC curve. (E) PR curve. (F) Ideal calibration curve. (G) Clinical decision curve. PR = precision–recall, ROC = receiver operating characteristic.

Using the boruta algorithm for variable selection, we identified 12 important attributes: age, height, weight, gender, race, arthritis, heart failure, coronary heart disease, heart attack, angina pectoris, emphysema, and cancer. Four attributes were deemed unimportant: hypertension, diabetes, chronic bronchitis, and liver condition (Table 4). These important variables were then subjected to LASSO regression analysis, and we created the LASSO regression variable selection path diagram and cross-validation plot (Fig. 3A and B). LASSO regression analysis was used to select variables for modeling analysis, and a nomogram was constructed (Fig. 3C). ROC curve and PR curve analysis were then performed, resulting in an AUC of 0.828 (Fig. 3D and E). The calibration curve and clinical decision curve were also used to validate the model’s performance (Fig. 3F and G).

**Table 4:** Variable selection with boruta algorithm.

Vars	Mean importance	Median importance	Min importance	Max importance	Norm hits	Final decision
Age	16.6572511	19.113073	8.1393880	23.141425	1.0000000	Confirmed
Heart failure	17.2906884	17.205996	13.9345974	21.516899	1.0000000	Confirmed
Gender	15.5750031	15.536359	13.6490412	17.505747	1.0000000	Confirmed
Coronary heart disease	15.1448701	15.151612	11.8603994	18.715136	1.0000000	Confirmed
Height	13.5385110	13.605036	11.5288897	15.861583	1.0000000	Confirmed
Weight	11.2772337	11.208177	8.1089953	15.526237	1.0000000	Confirmed
Heart attack	11.2030680	11.062792	8.4381271	13.877067	1.0000000	Confirmed
Arthritis	10.7140619	11.017958	5.7549592	16.439527	1.0000000	Confirmed
Angina pectoris	10.2957947	10.116624	7.8854853	12.922336	1.0000000	Confirmed
Emphysema	6.4259177	6.461859	1.9525234	9.407663	0.9642857	Confirmed
Cancer	5.3909676	5.268998	2.9482539	8.257765	0.9642857	Confirmed
Race	4.0362382	3.835027	0.9077896	7.936285	0.8214286	Confirmed
Chronic bronchitis	−0.7969129	−1.227008	−2.6474042	1.759569	0.0000000	Rejected
Diabetes	−3.8291277	−3.847705	−6.9913089	−1.090857	0.0000000	Rejected
Liver condition	−3.6793150	−4.078875	−5.1625960	−2.156523	0.0000000	Rejected
Hypertension	−6.1241435	−5.944534	−8.4412805	−4.874722	0.0000000	Rejected

Mean importance reflects the overall contribution level of variables to clinical prediction. Median importance resists the influence of extreme values and represents the predictive ability of variables. The low min importance of a certain variable indicates that it may be almost useless under certain modeling conditions. Max importance indicates that this variable is extremely critical to the model in certain situations.

## 4. Discussion

This article discusses the development of a stroke risk prediction model based on the NHANES database. The NHANES database was chosen for this study due to its broad representation and extensive health and nutrition data from a large population, making it ideal for such research. The study included 9922 patients, of whom 358 experienced a stroke, providing a sufficient sample size for robust statistical analysis. The baseline table emphasizes the differences in various variables between stroke patients and nonpatients, which are crucial for constructing predictive models.

Age is a significant risk factor for stroke.^[9]^ As individuals age, their blood vessels gradually deteriorate, and the risk of atherosclerosis rises. This can result in reduced or interrupted cerebral blood flow, thereby elevating the risk of stroke. Height may be related to blood pressure, and people with higher height may have higher blood pressure, which may lead to the occurrence of stroke, although the exact mechanism of this relationship is still unclear. The risk of having a stroke is elevated by obesity.^[10]^ Excess weight or obesity is associated with multiple cardiovascular diseases, such as hypertension, diabetes, and high cholesterol, all of which are risk factors for stroke.

Gender can affect the risk of stroke,^[11]^ and there are significant differences in risk factors and disease manifestations between genders. Hypertension is one of the most significant modifiable risk factors for stroke.^[12]^ Prolonged high blood pressure can damage the walls of blood vessels, increasing the risk of thrombosis and hemorrhage. Patients with diabetes often experience vascular damage and blood sugar control issues, increasing the risk of atherosclerosis and thrombosis, which in turn raises the likelihood of stroke. For example, a study conducted in Florida found that the prevalence of stroke in patients with diabetes was much higher than that in the general population.^[13]^

Patients with arthritis have a significantly increased risk of stroke and myocardial infarction due to an increase in systemic inflammation.^[14]^ Heart failure, characterized by weakened heart pumping function, can result in poor blood circulation, thereby elevating the risk of stroke. In a cohort study based on the national population, the long-term risk of all stroke subtypes in patients with heart failure is still higher than that in the general population.^[15]^ Coronary heart disease, a condition affecting the heart’s blood vessels, can lead to myocardial ischemia and heart dysfunction, increasing the risk of thrombosis and stroke. Angina pectoris, a symptom of myocardial ischemia, is often associated with coronary artery disease and can heighten the risk of stroke. Following a heart attack, heart function may be compromised, increasing the risk of thrombosis and stroke.

Emphysema is a major pathological feature of chronic obstructive pulmonary disease (COPD), and the relationship between COPD and cardiovascular disease has been extensively studied. There is literature research indicating that COPD patients have a significantly increased risk of stroke, which may be related to shared risk factors between COPD and stroke.^[16]^ In addition, research has found that symptoms of chronic bronchitis, such as long-term coughing and sputum production, are independently associated with the risk of stroke.^[17]^ Liver conditions can affect the body’s metabolic functions, including lipid metabolism, and may be closely related to the risk of atherosclerosis and cerebrovascular diseases. The link between cancer and stroke may be related to the hypercoagulable state caused by cancer. Cancer patients often exhibit abnormal blood clotting function, which may lead to thrombosis and increase the risk of stroke.^[18,19]^

Although asthma, chronic bronchitis, and arthritis are rarely mentioned in traditional stroke risk factors, previous studies have suggested that they may be associated with stroke risk (e.g., asthma and stroke share common risk factors and show some correlation).^[20]^ Rheumatoid arthritis has been found to independently increase the risk of stroke.^[21]^ Therefore, including it aims to comprehensively control possible confusion. In addition, the use of statins may play certain roles in primary and secondary prevention of stroke.

Some studies on modeling for predicting cerebrovascular outcomes have shown that machine learning models perform better than traditional models and achieve good AUC and accuracy in both internal and external validation cohorts.^[22]^ By drawing on similar methodological principles, our modeling framework has improved its reliability and is consistent with current best practices for predicting cerebrovascular outcomes. To ensure the robustness of the method, we used 3 different methods for selecting predictive variables: LASSO combined with stepwise regression, random forest and Boruta algorithm combined with LASSO regression. Our goal is to validate and optimize the predictive performance of the model using multiple complementary methods. This study emphasizes the value of combining different algorithms to improve prediction accuracy and generalizability.

In LASSO regression, a linear regression model that uses L1 regularization is able to automatically select variables, reduce model complexity, and enable variable selection automatically. Stepwise regression is an automated variable selection method where it increases and decreases variables iteratively until the best model can be found. In addition to the model-derived variables obtained by LASSO regression, stepwise regression analysis affirmed the statistical significance of these variables. The nomogram is a graph that shows the association of various factors with the prediction outcome. The chosen variables from this study were used to generate the nomogram model, by which the risk factors of stroke could be comprehended by doctors and patients to the fullest extent. The ROC curve is a beneficial tool for the evaluation of a machine learning model, and the AUC is a parameter that states the model’s ability to be discriminative. The PR curve, a great tool for dealing with imbalanced datasets, was applied to assess the precision and recall of the model for different thresholds. The calibration curve evaluated the model’s calibration performance and thus, measured the degree of conformity of the predicted probabilities to actual outcomes. The clinical decision curve that connects the calibration and discriminative abilities of the model made the assessment, across different clinical decision thresholds, and through its operational potential in practical clinical application. These improvements make the model highly suitable for clinical practice, which allows the use of the tool by doctors in assessing the risk a patient encounters for a stroke.

Random forest is an ensemble learning method that enhances model accuracy and robustness by constructing multiple decision trees and combining their predictions. It performs exceptionally well in handling large numbers of variables and nonlinear relationships and can evaluate the importance of each variable in predicting outcomes. This model performs well in terms of accuracy and AUC ROC score.^[23,24]^ Similar to the LASSO regression model, the random forest model also utilized the variables chosen to build the nomogram model. A nomogram is an intuitive method for showing the relationship of input variables to the prediction target, which is useful in informing patient and doctor decisions. The ROC curve examination of the random forest model presented the AUC of 0.612. Although the value is less than the AUC of the LASSO regression model (0.843), it demonstrates that the random forest model has some discriminating capability. The PR curve further confirmed the model’s performance, and the calibration curve and the clinical decision curve were used to underline its strong and weak points, respectively.

The boruta algorithm is a feature selection method based on random forests that assesses the importance of features by comparing their performance in the original dataset to a randomly perturbed dataset.^[25]^ The important variables identified by the boruta algorithm were further analyzed using the LASSO regression model to evaluate their predictive ability. Through the action of regularization term LASSO regression provides the opportunity to downgrade the model complexity and identify the variables that have the most significant effect on the prediction. The construction of var selection path diagram and cross-validation plot helped in evaluating the stability and accuracy of the LASSO regression model. To be able to have more inclusive piecewise mathematical nomogram modeling, the LASSO regression selected a few key options that were already created. The AUC value of 0.828 of the study indicators of a good or higher level of the model accuracy and discriminative power. Nonetheless, the PR curve does a better job explaining the model’s meatiness in practical applications. On the other hand, the calibration curve and clinical decision curve are helpful tools for researchers and clinicians to understand the model applicability in various clinical scenarios. There is a formulation that combines the boruta algorithm and the LASSO regression. This is a really good prediction model for stroke high risk people. The model is super important in detecting at-risk patients and preventing strokes.

Although the predictive performance of our model is moderate, this limitation highlights important avenues for further improvement. These comparisons indicate that while classical models maintain an advantage in interpretability, the machine learning methods used in this study have improved the handling of nonlinear relationships and variable selection. The observed performance gap suggests that data features such as sample size and variable quality may limit the accuracy of predictions. However, by systematically applying and evaluating multiple algorithms, our research provides a valuable framework for predictive modeling of NHANES data. Future research involves larger queues, more diverse variables, and advanced methods such as deep learning or ensemble techniques, which may further improve model performance and clinical applicability.

However, limitations do exist. Even though the random forest model showed some advantages in variable selection and prediction, it still had an AUC value that was not so high, so the model is not that predictive. This may have to do with the complexity of the model, the nature of the dataset, or the method of variable selection. On the other hand, LASSO regression accompanied with stepwise regression model performed well, and besides that, the subsequent studies should involve issues regarding additional variables and model types. There are other machine learning methods that could be combined with many different techniques to help predict the accuracy of the results. In addition, some external validation of the model is a necessary step to ensure its generalizability over different populations in the different settings.

## 5. Conclusions

In summary, the 3 models, LASSO combined with stepwise regression model, random forest, and LASSO algorithm associated with boruta algorithm, succeeded in the selection of variables, modeling, and performance evaluation in stroke risk prediction. Regardless of the fact that machine learning models still have to cover some gaps in their predictive abilities, they have established all related groundwork that can be built upon by further research and optimization. These models consist of the basis of future research and they key into the clinical practice.

## Acknowledgments

We express our gratitude to all NHANES participants and staff.

## Author contributions

**Conceptualization:** Junzhang Huang, Wencai Liu.

**Data curation:** Junzhang Huang.

**Methodology:** Junzhang Huang.

**Supervision:** Wencai Liu.

**Visualization:** Wencai Liu.

**Writing – original draft:** Junzhang Huang.

**Writing – review & editing:** Wencai Liu.

## References

[1] Shah B Bartaula B Adhikari J Neupane HS Shah BP Poudel G. Predictors of in-hospital mortality of acute ischemic stroke in adult population. J Neurosci Rural Pract. 2017;8:591–4. 29204020 10.4103/jnrp.jnrp_265_17 PMC5709883

[2] Chu H Li L Qiu B Tang Y. Editorial: outcomes of stroke: prediction and improvement. Front Neurol. 2023;14:1256253. 37560449 10.3389/fneur.2023.1256253 PMC10407940

[3] GBD 2019 Stroke Collaborators. Global, regional, and national burden of stroke and its risk factors,1990–2019: a systematic analysis for the global burden of disease study 2019. Lancet Neurol. 2021;20:795–820. 34487721 10.1016/S1474-4422(21)00252-0 PMC8443449

[4] Bailey RR. Promoting physical activity and nutrition in people with stroke. Am J Occup Ther. 2017;71:7105360010p1–5. 10.5014/ajot.2017.021378 PMC5557223 28809663

[5] Fernandes JND Cardoso VEM Comesaña-Campos A Pinheira A. Comprehensive review: machine and deep learning in brain stroke diagnosis. Sensors (Basel, Switzerland). 2024;24:4355. 39001134 10.3390/s24134355 PMC11244385

[6] Burgos N Colliot O. Machine learning for classification and prediction of brain diseases: recent advances and upcoming challenges. Curr Opin Neurol. 2020;33:439–50. 32657885 10.1097/WCO.0000000000000838

[7] Cui C Hong Y Bao J He L. The diagnostic reliability and validity of noninvasive imaging modalities to assess leptomeningeal collateral flow for ischemic stroke patients: a systematic review and meta-analysis. Medicine (Baltimore). 2021;100:e25543. 33950927 10.1097/MD.0000000000025543 PMC8104240

[8] Johnson AF Lamontagne N Bhupathiraju SN Brown AG Eicher-Miller HA. Workshop summary: building an NHANES for the future. Am J Clin Nutr. 2024;119:1075–81. 38331096 10.1016/j.ajcnut.2024.02.001 PMC11181347

[9] Sierra C Coca A Schiffrin EL. Vascular mechanisms in the pathogenesis of stroke. Curr Hypertens Rep. 2011;13:200–7. 21331606 10.1007/s11906-011-0195-x

[10] Koh IS Minn YK Suk SH. Body fat mass and risk of cerebrovascular lesions: the PRESENT (Prevention of stroke and dementia) project. Int J Environ Res Public Health. 2019;16:2840. 31398929 10.3390/ijerph16162840 PMC6721138

[11] Dehlendorff C Andersen KK Olsen TS. Sex disparities in stroke: women have more severe strokes but better survival than men. J Am Heart Assoc. 2015;4:e001967. 26150479 10.1161/JAHA.115.001967 PMC4608080

[12] Portegies ML Mirza SS Verlinden VJ. Mid- to late-life trajectories of blood pressure and the risk of stroke: the rotterdam study. Hypertension (Dallas, Tex. : 1979). 2016;67:1126–32. 27160196 10.1161/HYPERTENSIONAHA.116.07098

[13] Khan MM Roberson S Reid K Jordan M Odoi A. Prevalence and predictors of stroke among individuals with prediabetes and diabetes in Florida. BMC Public Health. 2022;22:243. 35125102 10.1186/s12889-022-12666-3 PMC8818177

[14] Kitas GD Gabriel SE. Cardiovascular disease in rheumatoid arthritis: state of the art and future perspectives. Ann Rheum Dis. 2011;70:8–14. 21109513 10.1136/ard.2010.142133

[15] Adelborg K Szépligeti S Sundbøll J. Risk of stroke in patients with heart failure: a population-based 30-year cohort study. Stroke. 2017;48:1161–8. 28377383 10.1161/STROKEAHA.116.016022

[16] Portegies ML Lahousse L Joos GF. Chronic obstructive pulmonary disease and the risk of stroke. The Rotterdam study. Am J Respir Crit Care Med. 2016;193:251–8. 26414484 10.1164/rccm.201505-0962OC

[17] Grau AJ Preusch MR Palm F Lichy C Becher H Buggle F. Association of symptoms of chronic bronchitis and frequent flu-like illnesses with stroke. Stroke. 2009;40:3206–10. 19679842 10.1161/STROKEAHA.109.561019

[18] Navi BB Iadecola C. Ischemic stroke in cancer patients: a review of an underappreciated pathology. Ann Neurol. 2018;83:873–83. 29633334 10.1002/ana.25227 PMC6021225

[19] Dardiotis E Aloizou AM Markoula S. Cancer-associated stroke: pathophysiology, detection and management (Review). Int J Oncol. 2019;54:779–96. 30628661 10.3892/ijo.2019.4669 PMC6365034

[20] Fang ZH Li ZF An ZY Huang SC Hao MD Zhang WX. Meta-analysis of the association between asthma and the risk of stroke. Front Neurol. 2022;13:900438. 35812117 10.3389/fneur.2022.900438 PMC9263265

[21] Al-Ewaidat OA Naffaa MM. Stroke risk in rheumatoid arthritis patients: exploring connections and implications for patient care. Clin Exp Med. 2024;24:30. 38294723 10.1007/s10238-023-01288-7 PMC10830780

[22] Cui C Lan J Lao Z Xia T Long T. Predicting the recurrence of spontaneous intracerebral hemorrhage using a machine learning model. Front Neurol. 2024;15:1407014. 38841700 10.3389/fneur.2024.1407014 PMC11150637

[23] Aboonq MS Alqahtani SA. Leveraging multivariate analysis and adjusted mutual information to improve stroke prediction and interpretability. Neurosciences (Riyadh, Saudi Arabia). 2024;29:190–6. 38981634 10.17712/nsj.2024.3.20230100 PMC11305345

[24] Alanazi EM Abdou A Luo J. Predicting risk of stroke from lab tests using machine learning algorithms: development and evaluation of prediction models. JMIR Formative Res. 2021;5:e23440. 10.2196/23440 PMC8686476 34860663

[25] Tang S Wang H Li K. C-reactive protein-triglyceride glucose index predicts stroke incidence in a hypertensive population: a national cohort study. Diabetol Metab Syndrome. 2024;16:277. 10.1186/s13098-024-01529-z PMC11580337 39574139

